# As Critical as the Score in the E‐Sports World: The Effect of Stress on Eating Behavior

**DOI:** 10.1002/fsn3.71628

**Published:** 2026-03-09

**Authors:** Ceren Şarahman Kahraman, Cansu Memiç İnan, Nurcan Yabancı Ayhan

**Affiliations:** ^1^ Department of Nutrition and Dietetics University of Alanya Alaaddin Keykubat Alanya/Antalya Turkey; ^2^ Department of Nutrition and Dietetics University of Hitit Çorum Turkey; ^3^ Department of Nutrition and Dietetics University of Ankara Ankara Turkey

**Keywords:** eating behavior, eating disorder, emotional eating, e‐sports, stress

## Abstract

In e‐sports players, the competitive environment and increased time spent in front of the screen for the game may lead to stress and physical inactivity, and individuals may develop negative eating behaviors. In this study, it was aimed to examine the relationship between perceived stress and eating behavior in e‐sports players. This cross‐sectional correlational study was conducted with 235 e‐sports players aged between 18 and 29 years who are members of the e‐sports community of a public university in Turkey. The questionnaire form used to obtain the study data included information on general and eating habits, Adult Eating Behavior Scale, SCOFF Eating Disorders Scale and Perceived Stress Scale. Among the e‐sports players who participated in the study, 85.5% were male and 50.2% were between the ages of 18–23 (mean age 23.9 ± 4.4). The results of regression analysis showed that as the perceived stress level increased in e‐sports players, SCOFF (*β* = 1.093), emotional over‐eating (*β* = 0.306), hunger (*β* = 0.455), and emotional under‐eating (*β* = 0.193) scores tended to increase (*p* < 0.05). Perceived stress level was found to be associated with eating behaviors in individuals interested in e‐sports. These results reveal the importance of interventions that support stress management and healthy eating behaviors for e‐sports players.

## Graphical abstract

Perceived stress affects eating behavior in e‐sports players.

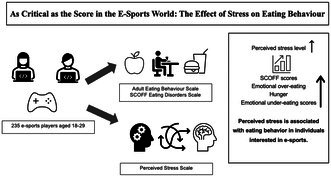

## 1 Introduction

Electronic sport (e‐sports), also known as video games or competitive games, is an entertainment activity in which one or more people compete in online/video games with a specific rule and is an increasingly popular in society (Formosa et al. 2022). It is stated that the number of individuals interested in e‐sports worldwide (regularly playing or watching) was 215 million in 2020, 234 million in 2021, and the number of regular video game players is higher (Gündoğdu et al. 2021). It is also predicted that 123.7 million more users may be added to these numbers between 2025 and 2029, and a new peak of 896.03 million users may be reached by 2029 (Statista 2025). The increasing social interest in e‐sports has led to an increase in academic studies in this field. In the literature, the economic aspect of e‐sports (Ströh 2017), its effects on brain functions (Shams et al. 2015; Stanmore et al. 2017) and psychological effects (González‐Bueso et al. 2018; Bányai et al. 2019) have been investigated, and its relationship with eating behavior has also started to be investigated in recent years (Ribeiro et al. 2023; Arslan et al. 2024).

General lifestyle interventions that can improve e‐sports performance include physical activity and sleep patterns as well as nutrition and eating behavior (AlMarzooqi et al. 2022; Goulart et al. 2023). In e‐sports, which requires a combination of motor and cognitive skills as well as strategy and imitation skills (van Hilvoorde and Pot 2016), eating behavior is very important for cognitive performance (Baumann et al. 2022). It is observed that individuals interested in e‐sports have lower vegetable and fruit consumption compared to daily recommendations, and long‐term gaming is associated with poor eating habits (Huth 2021; Chan et al. 2022). In addition, perceived stress reactions such as the thought of being enough in e‐sports, increased competition, nervousness, and excitement can lead to unhealthy life habits such as caffeine consumption, alcohol, and smoking (Pereira et al. 2021; Leis et al. 2022). E‐sports and the games played lead to insufficient physical activity with the increase in the time spent in front of the screen due to its nature, which makes individuals interested in e‐sports a risky population in terms of general health status and chronic diseases (Rudolf et al. 2020; Monteiro Pereira et al. 2022).

It is stated that one in four university students worldwide is overweight or obese, and the factors affecting this situation may be negative eating behaviors, poor mental health and high stress levels (Du et al. 2022). It is also known that higher stress levels lead to unhealthy eating habits, which are associated with higher energy and fat intake, more frequent sweets, and lower fruit and vegetable consumption (Zellner et al. 2006; Vidal et al. 2018). In a study, it was reported that stress management intervention to reduce the perceived stress level reduced the consumption of sweet snacks (Errisuriz et al. 2016). Common problems associated with e‐sports and gaming include increased stress levels (Smith et al. 2019). Similar to traditional sports, e‐sports players are reported to be exposed to stress as they are under pressure and compete in competitive environments (Palanichamy et al. 2020). In a systematic review, it was found that e‐sports with a competitive environment are associated with physiological and psychological stress, and that individuals' cortisol levels and anxiety levels increase from the beginning to the end of the game (Palanichamy et al. 2020). The increase in the time spent in front of the screen by individuals interested in e‐sports has also been associated with impaired stress regulation in individuals (Rudolf et al. 2022). In addition, in a study examining League of Legends (LoL) players, one of the competitive e‐sports, it was revealed that e‐sports players were exposed to various stressors due to competitive pressure, harassment by others, and negative communication during performance (Himmelstein et al. 2017).

Stress can increase cortisol levels in individuals, leading to stimulation of lipogenesis and altering hunger state and eating behaviors (Richardson et al. 2015). Perceived stress, which is called the subjective perception of stress, is known to be associated with eating behavior, especially emotional eating (Diggins et al. 2015; Shen et al. 2020). High perceived stress in university students led to emotional eating in both men and women (Du et al. 2022). When the high stress level in e‐sports players is combined with eating behaviors including unhealthy eating habits and a sedentary lifestyle, it is thought that these individuals may be at risk for various health problems. However, the limited number of studies on eating behaviors in individuals interested in e‐sports reveals the need for further research on this subject. Therefore, this study aimed to examine the relationship between perceived stress level and eating behaviors in individuals interested in e‐sports.

## 2 Materials and Methods

### 2.1 Participants

The sample of this study consists of e‐sports players who are 18 years old and over and who are members of the video games and electronic sports community of a public university in Turkey. In the calculation of the sample size for the study, it was determined that at least 233 individuals interested in e‐sports should be included, assuming a confidence interval of 90%, a margin of error of 5%, and an estimated medium effect size (*d* = 0.30), based on the study conducted by Arslan et al. (2024), using the G*Power program. Within the scope of the study, 272 e‐sports players who met the inclusion criteria and gave voluntary consent were reached. However, after excluding those with missing data and outliers, the remaining 235 e‐sports players constituted the sample.

Inclusion criteria for the study:

- ≥ 18 years of age.
- Being a student at university.
- Playing computer/video games (interested in e‐sports).
- Signing the voluntary consent form.

Exclusion criteria for the study:

- < 18 years of age.
- Having a chronic disease that may affect eating behavior (diabetes, hypertension, kidney diseases, etc.).
- Applying any disease‐specific diet.
- Previous history of eating disorders (anorexia, bulimia, etc.).
- Use of psychiatric medication.

### 2.2 Measures

#### 2.2.1 Questionnaire Form

The study data were collected through a questionnaire form using face‐to‐face interview technique. In the questionnaire form, in addition to general information of the individuals, questions to determine some eating habits, Adult Eating Behavior Scale, SCOFF Eating Disorders Scale and Perceived Stress Scale were included. In the general information section of the questionnaire form, in addition to questions about the age, gender, marital status, educational status, income level, smoking and alcohol consumption of the participants, questions such as the number of main and intermediate meals, skipping meals, snack consumption, water/energy drink/tea/coffee consumption to determine their eating habits, questions about e‐sports related player status, the type and name of the game played, the time spent and how long they have been interested in e‐sports were included. The physical activity status of the participants was evaluated with a multiple‐choice question as “how many days a week do you do physical activity for not less than 30 min at a time.”

#### 2.2.2 Adult Eating Behavior Scale

In order to determine in which situations food intake is triggered in adult individuals, the “Adult Eating Behavior Scale” developed by Hunot et al. (2016) and the validity and reliability of which was performed by Yücel et al. (2022) was used. The scale, which originally consisted of 35 items and 8 dimensions (hunger, food responsiveness, emotional over‐eating, enjoyment of food, satiety responsiveness, emotional under‐eating, food fussiness, slowness in eating), is scored on a 5‐point Likert scale (1 = never, 2 = rarely, 3 = sometimes, 4 = often, 5 = always). In the original scale, four items (12, 14, 19, 24) were reverse coded. The Turkish version of the scale used in our study consists of 26 items and 7 dimensions (hunger, satiety responsiveness, emotional over‐eating, enjoyment of food, emotional under‐eating, food fussiness and slowness in eating). There are items 5, 8, 10, 16, and 21 in the emotional over‐eating dimension, items 9, 28, 32, and 34 in the hunger dimension, items 15, 18, 20, 27, and 35 in the emotional under‐eating dimension, items 11, 23, and 31 in the satiety responsiveness dimension, items 12, 19, and 24 in the food fussiness dimension, items 1, 3, and 4 in the enjoyment of food dimension and items 25, 26, and 29 in the slowness in eating dimension. Although it is scored on a 5‐point Likert scale as in the original form, there are no reverse coded items in the Turkish form. The Cronbach α value of the Turkish form is 0.76. It is stated that the scale can be used to prevent various health problems by providing a better understanding of eating behaviors in adults. The increase in the score obtained from each sub‐dimension reflects the predisposition to the eating behavior in the sub‐dimension examined. In this study, the internal consistency of the scale was found to be Cronbach alpha = 0.816.

#### 2.2.3 SCOFF Eating Disorders Scale

In order to screen eating disorders, the “SCOFF Eating Disorders Scale” developed by Morgan et al. (1999) and the Turkish validity and reliability of which was conducted by Aydemir et al. (2015) was used. The Cronbach's *α* value of the Turkish form of the scale, which consists of 5 items and one dimension in total, is 0.74. Each item that fits the individual in the scale is given 1 point and the maximum score that can be obtained is 5. Individuals who score 2 and above on the scale are considered as a risk group for eating disorders. In this study, the Cronbach *α* reliability coefficient for the SCOFF Eating Disorders Scale was found to be 0.720.

#### 2.2.4 Perceived Stress Scale

In order to measure the extent to which individuals perceive various situations in their lives as stressful, the “Perceived Stress Scale” developed by Cohen et al. (1983) and the Turkish validity and reliability of which was conducted by Eskin et al. (2013) was used. This 5‐point likert‐type scale (never = 0, almost never = 1, sometimes = 2, fairly often = 3, very often = 4) consists of 14 items and 2 dimensions: perception of inadequate self‐efficacy and perception of stress/discomfort. Items 4, 5, 6, 8, 9, 10, and 13 are included in the dimension of perception of inadequate self‐efficacy, and items 1, 2, 3, 7, 11, 12, and 14 are included in the dimension of perception of stress/discomfort. The 7 questions with positive meanings (items 4, 5, 6, 7, 9, 10, and 13) are reverse coded. Scores between 0 and 56 can be obtained from the scale and the Cronbach's *α* value of the scale is 0.84. High scores on the scale indicate that the person's stress perception is high. The Perceived Stress Scale demonstrated good internal consistency in this study (Cronbach *α* = 0.841).

#### 2.2.5 Anthropometric Measurements

Information on the body weight (kg) and height (cm) of the individuals included in the study was obtained based on their declarations. Body mass index (BMI) was calculated by dividing body weight (kg) by the square of height (m)^2^. BMI classification according to WHO criteria: 18.50–24.99 kg/m^2^ is considered normal, 25.0–29.99 kg/m^2^ is considered overweight, and ≥ 30.00 kg/m^2^ is considered obese (WHO 2020).

### 2.3 Data Analysis

Statistical Package for the Social Sciences (SPSS) 26 package program was used for statistical analysis. The compatibility of the variables in the data set with normal distribution was evaluated with Kolmogorov–Smirnov/Shapiro–Wilk tests, skewness‐ kurtosis and histogram curves and it was determined that the data were normally distributed in the analyzes. Descriptive data are presented in number‐percentage tables. Variables are presented as mean and standard deviation. In the evaluation of categorical variables, Pearson chi‐square test was used when the expected frequency value was less than 0.20, and Fisher's exact test was used when the expected frequency value was greater than 0.20. The significance of the difference between the two groups was determined using an independent samples *t*‐test. The correlation between numerical variables was determined by Pearson correlation test. Linear regression model was used to determine the variables affecting perceived stress. All analyses were evaluated at 0.05 significance level.

### 2.4 Ethical Considerations

Before collecting the data, ethics committee permission dated 10.09.2024 and decision numbered 2024/14 was obtained from Alanya Alaaddin Keykubat University Non‐Interventional Clinical Research Ethics Committee. The study was conducted in accordance with the Declaration of Helsinki and voluntary consent was obtained from all participants.

## 3 Results

General information of the individuals participating in the study is given in Table 1. 85.5% of the participants were male, 50.2% were between the ages of 18–23 (mean age 23.9 ± 4.4), and 82.6% were associate's or bachelor's degree graduates. Those with low (41.3%) and high (42.1%) incomes were similar, with the majority of participants consuming cigarettes (52.8%) and alcohol (56.2%). 46.8% of the participants had normal body weight (mean BMI 25.5 ± 4.6) and 39.6% engaged in physical activity 1–2 days a week. According to e‐sports classification, 54% of individuals are regular players and 76.1% play player versus player (PVP) (Table 1).

**TABLE 1:** General information of participants (*n* = 235).

		*n* (%)
Sex	Male	201 (85.5)
	Female	34 (14.5)
Age groups^a^	18–23	118 (50.2)
	24–29	117 (49.8)
Education status	Associate‐Bachelor's degree	194 (82.6)
	Postgraduate	41 (17.4)
Income	Low	97 (41.3)
	Medium	39 (16.6)
	High	99 (42.1)
Marital status	Single	17 (7.2)
	Married	218 (92.8)
Smoking	Yes	124 (52.8)
	No	111 (47.2)
Alcohol	Yes	132 (56.2)
	No	103 (43.8)
BMI classification	Underweight (< 18.5 kg/m^2^)	9 (3.8)
	Normal (18.5–24.99 kg/m^2^)	110 (46.8)
	Overweight (25.0–29.99 kg/m^2^)	84 (35.7)
	Obese (≥30 kg/m^2^)	32 (13.6)
Frequency of physical activity	Everyday	17 (7.2)
	5–6 days a week	16 (6.8)
	3–4 days a week	41 (17.4)
	1–2 days a week	93 (39.6)
	Never	68 (28.9)
E‐sport classification	Professional	29 (12.3)
	Amateur	79 (33.6)
	Regular player	127 (54.0)
Type of game played	Player vs. player (PVP)	179 (76.1)
	Player vs. enemy (PVE)	56 (23.9)
Duration of interest in e‐sports	1–3 years	115 (48.9)
	4–6 years	47 (20.0)
	7 years and above	73 (31.1)
Number of main meals	One	9 (3.8)
	Two	113 (48.1)
	Three	113 (48.1)
Snack consumption	Nuts	131 (21.1)
	Sweets/dessert/chocolate	113 (18.2)
	Junk food	132 (21.3)
	Patisserie products	31 (5.0)
	Fastfood	76 (12.3)
	Fruit and vegetables	81 (13.1)
	Coffee	5 (0.8)
	Dairy products	51 (8.2)
		$\bar{X}$ **± SD**
Age (year)		23.9 ± 4.4
BMI (kg/m^2^)		25.5 ± 4.6
Time spent with the game (h/day)		4.6 ± 2.8

^a^ Age was classified based on the median value.

The most popular games played by the participants were League of Legends (LoL) with 27.0%, Valorant with 18.1%, and Counter‐Strike: Global Offensive with 17.0% (not shown in the table). The majority of the individuals participating in the study have been interested in e‐sports for 1–3 years and spend an average of 4.6 ± 2.8 h a day playing the game. The majority of participants eat two or three meals. The most frequently skipped meal was breakfast (46%), followed by lunch (26.8%) (not shown in the table). The most common snacks consumed by the participants were junk food (21.3%) and nuts (21.1%) (Table 1).

Table 2 presents the study variables according to the perceived stress level classification. It was determined that the duration of interest in e‐sports, time spent with the game (h/day), SCOFF score, emotional over‐eating score, and hunger score showed a significant difference according to the perceived stress level classification (*p* < 0.05). It was found that the frequency of those who were interested in e‐sports for 1–3 years, time spent with the game, SCOFF score, emotional over‐eating score, and hunger score was statistically significantly higher in individuals with high perceived stress level (*p* < 0.05) (Table 2).

**TABLE 2:** Various variables according to perceived stress level.

	Classification of perceived stress level		*p*
	Low	High	
Sex			
Male	16 (13.2)	18 (15.8)	0.576
Female	105 (86.8)	96 (84.2)	
Age groups			
18–23	58 (47.9)	60 (52.6)	0.472
24–29	63 (52.1)	54 (47.4)	
Education status			
Associate‐Bachelor's degree	99 (81.8)	95 (83.3)	0.760
Postgraduate	22 (18.2)	19 (16.7)	
Income			
Low	48 (39.7)	49 (43.0)	0.534
Medium	18 (14.9)	21 (18.4)	
High	55 (45.5)	44 (38.6)	
BMI classification			
Underweight and normal (< 25 kg/m^2^)	67 (55.4)	52 (45.6)	0.135
Overweight and obese (≥ 25 kg/m^2^)	54 (44.6)	62 (54.4)	
E‐sport classification			
Professional	16 (13.2)	13 (11.4)	0.055
Amateur	32 (26.4)	47 (41.2)	
Regular player	73 (60.3)	54 (47.4)	
Duration of interest in e‐sports			
1–3 years	50 (41.3)	65 (57.0)	**0.005**
4–6 years	22 (18.2)	25 (21.9)	
7 years and above	49 (40.5)	24 (21.1)	
Type of game played			
Player vs. player (PVP)	90 (74.4)	89 (78.1)	0.507
Player vs. enemy (PVE)	31 (25.6)	25 (21.9)	
Time spent with the game (h/day)	4.2 ± 2.4	5.1 ± 3.2	**0.019**
Age (years)	24.1 ± 4.6	23.8 ± 4.1	0.596
BMI (kg/m^2^)	25.3 ± 4.8	25.8 ± 4.3	0.372
SCOFF score	0.8 ± 1.1	1.2 ± 1.1	**0.006**
Emotional over‐eating	9.8 ± 4.7	13.0 ± 5.9	**< 0.001**
Hunger	9.8 ± 3.4	11.8 ± 3.9	**< 0.001**
Emotional under‐eating	13.7 ± 6.2	14.3 ± 5.4	0.464
Satiety responsiveness	7.4 ± 2.6	7.8 ± 3.0	0.339
Food fussiness	11.3 ± 2.7	11.6 ± 3.1	0.468
Enjoyment of food	11.3 ± 2.3	11.4 ± 2.8	0.827
Slowness in eating	7.2 ± 3.3	7.5 ± 3.1	0.535

*Note:* Perceived stress level was classified as low and high based on the median value. Chi‐square test was conducted for categorical variables, and independent sample *t*‐test was applied for nominal variables. Bold values show statistical significance. *p* < 0.05.

Table 3 shows the relationship between perceived stress level and eating behavior sub‐dimensions and eating disorders. Perceived stress level showed significant positive correlations with the scores of SCOFF (*r* = 0.266, *p* < 0.001), emotional over‐eating (*r* = 0.342, *p* < 0.001), hunger (*r* = 0.372, *p* < 0.001), and emotional under‐eating (*r* = 0.142, *p* = 0.029). In addition, a significant positive correlation was observed between perceived stress level and time spent in the game (*r* = 0.222, *p* = 0.001) (Table 3).

**TABLE 3:** The relationship between perceived stress level and eating behavior sub‐dimensions, eating disorder and time spent with gaming (h/day).

Variables	*r*	*p*
SCOFF score	0.266	**< 0.001**
Emotional over‐eating	0.342	**< 0.001**
Hunger	0.372	**< 0.001**
Emotional under‐eating	0.142	**0.029**
Satiety responsiveness	−0.054	0.408
Food fussiness	0.108	0.099
Enjoyment of food	0.118	0.071
Slowness in eating	0.016	0.810
Time spent with the game (h/day)	0.222	**0.001**

*Note:* Pearson correlation test was performed. Bold values show statistically significant. *p* < 0.05.

Information on some variables related to perceived stress level is given in Table 4. When the overall explanatory power of the model is analyzed, it is seen that *R* ^2^ = 0.285 and the model explains approximately 28.5% of the dependent variable, perceived stress (*p* < 0.001). SCOFF score (*β* = 1.093, 95% CI: 0.121–2.066, *p* = 0.023), emotional over‐eating score (*β* = 0.306, 95% CI: 0.070–0.542, *p* = 0.011), hunger score (*β* = 0.455, 95% CI: 0.094–0.816, *p* = 0.014) and emotional under‐eating score (*β* = 0.193, 95% CI: 0.009–0.377, *p* = 0.040), perceived stress level also tended to increase (Table 4).

**TABLE 4:** Linear regression analysis of associated factors with the perceived stress.

	Perceived stress score			
	Beta	95% CI		*p*
		Min.	Max.	
**Model 2**				
SCOFF score	1.093	0.121	2.066	**0.023**
Emotional over‐eating	0.306	0.070	0.542	**0.011**
Hunger	0.455	0.094	0.816	**0.014**
Emotional under‐eating	0.193	0.009	0.377	**0.040**
Satiety responsiveness	−0.287	−0.704	0.130	0.176
Food fussiness	0.094	−0.254	0.441	0.596
Enjoyment of food	−0.271	−0.725	0.183	0.241
Slowness in eating	0.032	−0.299	0.364	0.847

*Note:* Bold values show statistically significance. The model was adjusted for age, sex, marital status, income, education level, physical activity, time spent playing games (h/day), and BMI. Linear regression analysis was performed. *p* < 0.05. *p* < 0.001, *R* ^2^ effect size = 0.285.

## 4 Discussion

Despite the increasing interest in e‐sports all over the world, research on the health status of e‐sports players is limited (Trotter et al. 2020) and e‐sports players are known to have psychological problems, especially stress and anxiety, sedentary lifestyle, high BMI and negative nutritional behaviors that may negatively affect their health status (Shulze et al. 2021). In this study, which was conducted to determine the relationship between perceived stress and eating behavior in individuals interested in e‐sports, most of the participants (85.5%) were male, with a high level of education (82.6%) and young adults between the ages of 18–23 (50.2%). In traditional sports, men have a physical advantage over women, whereas in e‐sports, physical characteristics are not associated with high performance. This puts both male and female gender in an equal position in terms of e‐sports, but it is observed that the e‐sports industry is male‐dominated, with approximately 35% of e‐sports players and 5% of professional players being women (Rogstad 2021). In addition, it is stated that young people are at the forefront in terms of interest in e‐sports and active players, and in Norway, e‐sports is called “youth sports 2.0” and has been included in educational curricula and integrated into local sports clubs in recent years (Tjønndal and Skauge 2021). In a study conducted annually in France since 2018, it has been reported that e‐sports players are predominantly young individuals between the ages of 15–34 (91.0%) and men (93.0%) (Mediametrie, Baromètre France Esports 2023). In a cross‐sectional study examining 1066 individuals playing e‐sports and video games in Germany, it was found that 91.9% of the participants were male, 54.1% were regular players, the average age ranged between 20 and 30 years, the educational level of the participants was high and 49.0% of them played Counter‐Strike: Global Offensive (Rudolf et al. 2020). In another study examining the media use, stress and well‐being of video game and e‐sports players in Germany, it was observed that 91.2% of 1038 players were male and the average age was 23.0 ± 5.4 years (Rudolf et al. 2022). In line with the results of the previous studies, in this study, it is seen that individuals interested in e‐sports are predominantly young adults with high educational status and men. The fact that the data were taken from e‐sports community members studying at a public university affected the average age and education status.

Spending time playing games after long class hours at school or for 3–4 h after school may increase the time spent sitting, leading to insufficient physical activity and increasing the risk of chronic diseases (Bailey et al. 2019). It is known that increased screen time is associated with unhealthy lifestyles and increased rates of obesity (Marker et al. 2022). Arnaez et al. (2018) found that increased sitting time in adults who play games increases the likelihood of being obese by 2.69 times. As in traditional sports, e‐sports includes amateur and professional levels, and the weekly playing time varies according to the level of the player (Steinkuehler 2019). It was found that the weekly playing time was 28.6 ± 12.0 h in professional players, 28.4 ± 16.6 h in amateur players, and 21.7 ± 14.7 h in regular players (Rudolf et al. 2020). In this study, it was found that 54% of the participants were regular players and spent 4.6 ± 2.8 h daily in the game, and 39.6% of them performed physical activity for 30 min 1–2 days a week. This shows that the participants did not meet the WHO recommendation of 2.5 h of physical activity per week (WHO 2010). E‐sports players are reported to be inactive during training for approximately 4.2 h per day, with the exception of professional e‐sports players, who are reported to exercise and be physically active for up to 1 h per day as part of their training (Kari and Karhulahti 2016).

The mean BMI of the individuals participating in this study was found to be 25.5 ± 4.6. Rudolf et al. (2020) determined that 66.9% of individuals who played e‐sports and video games performed more than 2.5 h of moderate‐to‐vigorous physical activity per week, the daily sedentary time was 7.7 ± 3.6 h, the average sleep time during the night was 7.1 ± 1.3 h, and 51.3% of the participants had normal BMI. Rudolf et al. (2022) found that the mean BMI of e‐sports players was 24.8 ± 5.0 kg/m^2^. It was found that the duration of playing video games in a single sitting was positively associated with BMI and negatively associated with exercise frequency in male undergraduate students, and these relationships were stronger in those who played online games (Ballard et al. 2009). It is stated that the BMI of e‐sports players can vary between 23.1 and 26.0 kg/m^2^, so players are unlikely to be at a healthy weight or obese (Rudolf et al. 2020). In contrast, Trotter et al. (2020) found that e‐sports players were more likely to be categorized as normal weight or Grades 2 and 3 obese, the time spent playing e‐sports increased as the in‐game ranking of the e‐sports player increased, a small group of e‐sports players were obese, and most e‐sports players did not meet physical activity recommendations. According to the results of the study, the time spent in the game may be lower than that of a professional e‐sports player due to the fact that the participants are regular players, but in general, the low physical activity levels of individuals interested in e‐sports may be related to the BMI average of ≥ 25 kg/m^2^.

It is stated that spending long hours in e‐sports games is associated with negative eating behaviors such as inadequate and unbalanced nutrition and night eating (Cha et al. 2018). In addition, it has been reported that playing video games in men is associated with skipping meals, and individuals who play video games at least four times a week are more likely to skip meals compared to those who never play video games (Brooks et al. 2016). In a study examining the dietary habits and gaming behaviors of Portuguese and Brazilian e‐sports players, it was determined that one‐quarter (23.0%) of the participants (*n* = 579) never ate breakfast and almost half (47.5%) skipped breakfast 3 or more days a week (Ribeiro et al. 2023). Arslan et al. (2024) found that the most frequently skipped meal in e‐sports players was breakfast (41.9%). In our study, it was determined that 48.1% of e‐sports players had two meals and 46% of them skipped breakfast.

Emotional over‐eating refers to increased food consumption in response to negative emotions and stress and is a method of emotion regulation (Bemanian et al. 2020). Stress‐inducing and unpredictable changes can trigger emotional over‐eating (van Strien 2018). It is stated that emotional over‐eating is generally associated with the consumption of foods containing high energy, fat, and sugar, and this negatively affects the health status of individuals (Oliver et al. 2000). In our study, it was determined that the foods that e‐sports players frequently consume as snacks are junk food, nuts, and sweet/desert/chocolate. Szot et al. (2022) found that 233 Polish male e‐sports players aged 18–26 years exhibited unhealthy eating behaviors such as irregular eating and frequent snacking; 54.9% of the individuals consumed sweet/sugary foods, 28.3% fast food, 28.7% salty snacks, and only 2.7% energy drinks. Individuals' tendency to consume snacks may be due to skipping meals or low quality of the foods consumed.

University students are exposed to a wide range of stressors influenced by a developmental change process such as transition from adolescence to adulthood, academic and financial difficulties, and emotional situations outside the classroom (Graves et al. 2021). In addition, it is expected that the perceived stress in university students interested in e‐sports, which has a competitive environment, will be high. In our study, it was found that individuals with high perceived stress levels had a shorter period of interest in e‐sports (1–3 years) and more time spent in the game. Lissak (2018) reported that increased screen time is associated with decreased stress management, that is, higher stress levels. In a study evaluating mental fatigue and stress in e‐sports players, it was found that playing games increased stress and mental fatigue (Gündoğdu et al. 2021). It is expected that professional e‐sports players spend a significant amount of their time playing games as it is their livelihood. However, regular gamers also spend more than two to three hours a day playing games, which can negatively affect their general health (Rudolf et al. 2022). Being interested in e‐sports for a short period of time, that is, just starting e‐sports, may have led to an increase in the time spent in the game to train skills in regular or amateur players, as well as more stress perceived to achieve success during the game.

In order for e‐sports players to achieve a successful performance, they need to have knowledge about games, think strategically, act quickly and rationally, have high motivation, manage distraction, and manage stress well by displaying a positive attitude (Palanichamy et al. 2020). Excessive game playing leads to social and emotional problems, especially psychological distress (Saquib et al. 2017), and it is stated that psychological factors, especially stressful events, are one of the underlying causes of eating behavior disorders (Solmi et al. 2021). Arslan et al. (2024) reported that e‐sports players are a risky population in terms of eating disorders. Various studies have reported that disordered eating behavior and eating disorders are more frequently observed in athletes (Du et al. 2022; Kussman and Choo 2024). In this study, increases in the SCOFF score, emotional over‐eating, hunger, and emotional under‐eating scores were found to be significantly associated with an increase in perceived stress level. This finding supports the effect of stress on individuals' eating behaviors. In particular, the fact that both excessive and inadequate forms of emotional eating behaviors are associated with stress suggests that stress may have a bidirectional effect on eating behavior. The fact that the SCOFF score was a significant predictor indicates that risky eating behaviors should be evaluated together with stress in individuals interested in e‐sports. In contrast, behaviors such as satiety responsiveness, food fussiness, enjoyment of food, and slowness in eating were not significantly associated with stress, suggesting that stress particularly affects more impulsive and emotional eating patterns. In the literature, there is no study evaluating the relationship between perceived stress and eating behavior in e‐sports players. In this respect, this study is one of the pioneering studies revealing the relationship between psychology and eating behavior in individuals interested in e‐sports. The findings obtained may guide the development of holistic health approaches for this population.

The study has various limitations. Firstly, since this study is of descriptive correlational type, a causality relationship could not be determined. Another limitation is that anthropometric data such as body weight and height were obtained based on self‐reports. The fact that the sample consists of participants who study at a state university in Turkey and are members of an e‐sports community may have prevented the generalizability of the results to all e‐sports players in our country. On the other hand, the fact that there are limited studies examining the eating behavior of e‐sports players in our country and that our study is the first study evaluating the relationship between stress and eating behavior in e‐sports players in our country constitutes a unique value. In addition, this study may provide a different perspective to the studies to be planned to improve the health status of e‐sports players.

## 5 Conclusion

As one of the pioneering studies examining the relationship between perceived stress level and eating behaviors in individuals interested in e‐sports, this study revealed important results. The results of the study showed that eating disorders, emotional over‐eating, hunger, and emotional under‐eating scores increased significantly with increasing perceived stress level. This suggests that stress level may be related to eating behaviors in individuals interested in e‐sports. These results indicate that e‐sports players should be evaluated not only in terms of physical but also psychological and nutritional health risks. Therefore, there is a need for interventions that support stress management and healthy eating behaviors in individuals interested in e‐sports. In future studies, it is recommended to examine causal relationships by taking into account different age groups with larger samples.

## Author Contributions

**Ceren Şarahman Kahraman:** conceptualization (equal), data curation (equal), investigation (equal), methodology (equal), writing – original draft (equal). **Cansu Memiç İnan:** conceptualization (equal), data curation (equal), formal analysis (equal), investigation (equal). **Nurcan Yabanci Ayhan:** methodology (equal), writing – review and editing (equal).

## Funding

The authors have nothing to report.

## Ethics Statement

This study was conducted according to the guidelines laid down in the Declaration of Helsinki and all procedures involving research study participants were approved by the “Research Board Approval” was obtained from the Alanya Alaaddin Keykubat University Non‐Invasive Clinical Research Ethics Committee with the decision numbered 2024/14 and dated 10.09.2024. Informed consent was obtained from all participants included in the study. [Correction added on 24 March 2026, after original online publication: Statement updated to correct the decision number and approval date.]

## Conflicts of Interest

The authors declare no conflicts of interest.

## Data Availability

The data that support the findings of this study are available from the corresponding author upon reasonable request.
